# Adopting or Avoiding? Older Adults’ Ride-Hailing Perceptions and Experiences

**DOI:** 10.1093/geroni/igaf122.4060

**Published:** 2025-12-31

**Authors:** Paula Carder, Minju Song

**Affiliations:** Portland State University, Portland, Oregon, United States; Portland State University, Portland, Oregon, United States

## Abstract

This qualitative study explored how older adults perceive ride-hailing services and what factors influence their willingness to use them. Informed by the “accessibility framework,” we explored how individual, transport, land-use, temporal, and social-context factors influence older adults’ perceptions of and decisions about adopting services like Lyft and Uber. We conducted semi-structured interviews with 18 older adults (ages 66-83) in the Portland metropolitan region. Participants included 6 men and 12 women; 8 had never used ride-hailing, while 10 had done so at least once. Most identified as non-Hispanic White, with two Korean immigrants and one Chinese American. Interview questions included ride-hailing experiences and thoughts in terms of geographical, physical/psychological, and technological accessibility, as well as their transportation needs and the relationship between ride-hailing and public transit. All interviews were audio-recorded, transcribed, loaded into Atlas.ti software, and coded to identify common themes and positive and negative perspectives regarding ride-hailing. Participants’ perceptions and prior experiences informed decisions about ride-hailing use. Primary themes included concerns about safety at different times of the day, relative affordability, relative affordability compared to public transit or owning a vehicle, and difficulties with ride-hailing app usability and smartphone access. Participants had suggestions for making ride-hailing services more accessible to older adults and people with disabilities, and viewed ride-hailing as a complement or substitute to other transportation modes. Findings suggest that even when ride-hailing services are available, older adults may not adopt services if they do not perceive them as safe, affordable, easy to use, or physically accommodating.

